# Grade 3 invasive breast cancer masquerading as a haematoma — the importance of concordance in the clinical and imaging pathway

**DOI:** 10.1259/bjrcr.20220156

**Published:** 2023-03-17

**Authors:** Ramona Menezes, Daniella Kostic, Penelope Moyle

**Affiliations:** 1 Nottingham University Hospitals NHS Trust, England, UK; 2 Cambridge University Hospitals NHS Foundation Trust, England, UK

## Abstract

Cystic lesions of the breast are an everyday encounter in the symptomatic breast clinic. While the vast majority of cystic lesions are benign, it is important to be aware of the imaging manifestations that indicate a sinister pathology and the pitfalls of biopsy in a complex cystic lesion which make the diagnosis challenging. We present a case of cystic Grade 3 breast cancer and highlight the imaging characteristics and clinicoradiological concordance that achieved the correct diagnosis.

## Clinical presentation

A 46-year-old female attended the breast unit with a right breast lump, which had been present for 1 month. The lump had been increasing in size and pain over this time period.

Four months prior, she had fallen in her bathroom and onto a child’s step stool on her right side. She had pain on her right side but no bruising on her breast at that time. She was clinically fit and well, not on any medication with no family history of breast cancer. Clinical examination revealed a 45 mm tense lump in the right upper outer quadrant scored indeterminate (E3)^1^ with a normal axillary examination.

## Imaging findings/Investigations

**Figure 1.**
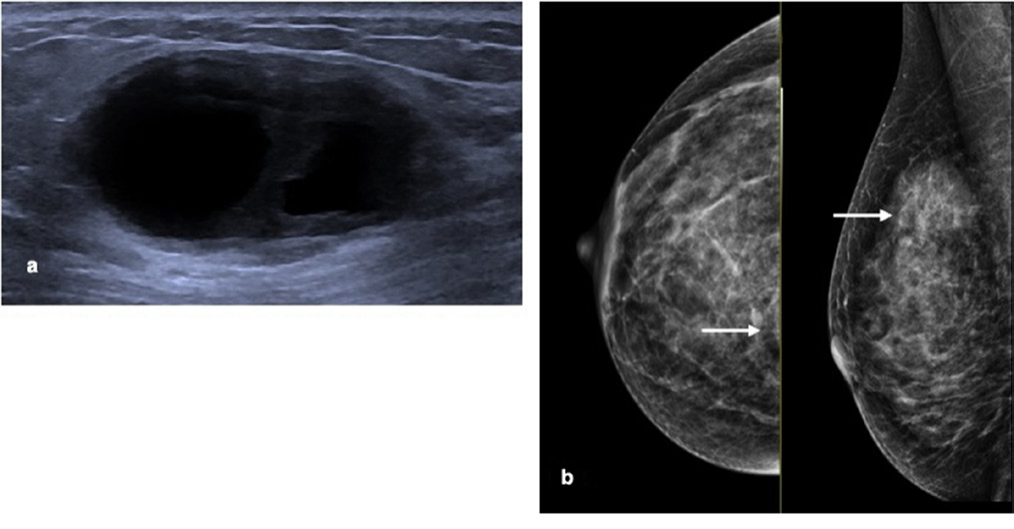
A. Ultrasound image shows a thick-walled complex cystic mass at site of palpable lump in the right superior breast at initial presentation. The presence of a thick wall and thick septations are features that indicate BI-RADS 4 characterisation and a biopsy is indicated. B: Mammography identifies a well-defined low density rounded mass in the right upper inner breast. This is medial and is only partially seen on the CC view (arrow). High medial lesions are often difficult to be seen on mammography.

A mammogram demonstrated a well-defined low density mass, measuring 35 mm in the upper medial right breast (M3)^2^ (Figure 1A). Ultrasound of the right breast confirmed a 33 mm well-defined, oval, fluid-filled mass in the upper medial breast. It had a slightly thickened wall, internal echogenicities and posterior acoustic enhancement (U3)^2^ (Figure 1B). Given her recent trauma history, it was thought that this could represent a haematoma, however, there was no clear correlation of direct trauma in this area and therefore it was decided to proceed with a biopsy (3 × 14G cores). The lesion partly collapsed and haemorrhagic fluid was drained from the residual lesion.

Histology of the core biopsy showed fibrosis and chronic inflammation with secondary reactive changes (B2). The patient was reassured and discharged from clinic.

**Figure 2.**
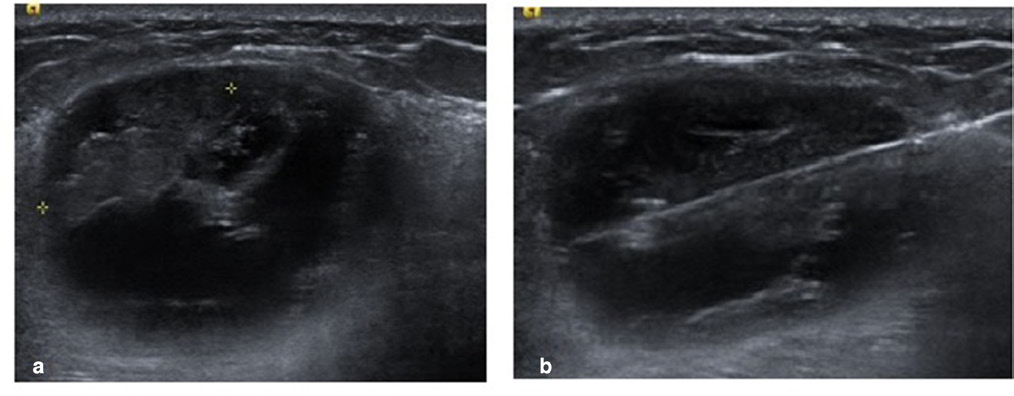
(A) Six weeks later, the patient returned with refilling of the lump. Ultrasound showed a large complex cystic mass with a solid intracystic nodule from its anterior aspect. (B) Ultrasound-guided core biopsy through the solid component showing good needle position.

Six weeks later, the patient re-presented to the breast unit with the same lump, which had increased in size. She denied any subsequent trauma to the area. Clinical examination demonstrated a large soft lump in the upper right breast (E2). Ultrasound of the recurrent mass (Figure 2A) revealed that it had increased in size to 42 mm. It was well-defined, oval, predominantly fluid-filled, although in the upper inner aspect there was a new lobulated hyperechoic solid component, which appeared to be arising from the anterior wall of the mass. This solid component measured 26 mm and was radiologically suspicious (U4). Core biopsy of the solid component (2 × 14G cores) was taken (Figure 2B) and the cystic component aspirated as much as possible (15 ml of blood stained fluid) with residual non-fluid hyperechoic soft tissue persisting.

The histopathology identified necrotic material containing two tiny clusters of partially degenerate epithelial cells. The report concluded that whilst this could represent a necrotic papillary lesion, the possibility of malignancy could not be excluded and it was best regarded as non-diagnostic. This case was reviewed at the Breast Multidisciplinary meeting and the recommendation was for surgical excision.

**Figure 3.**
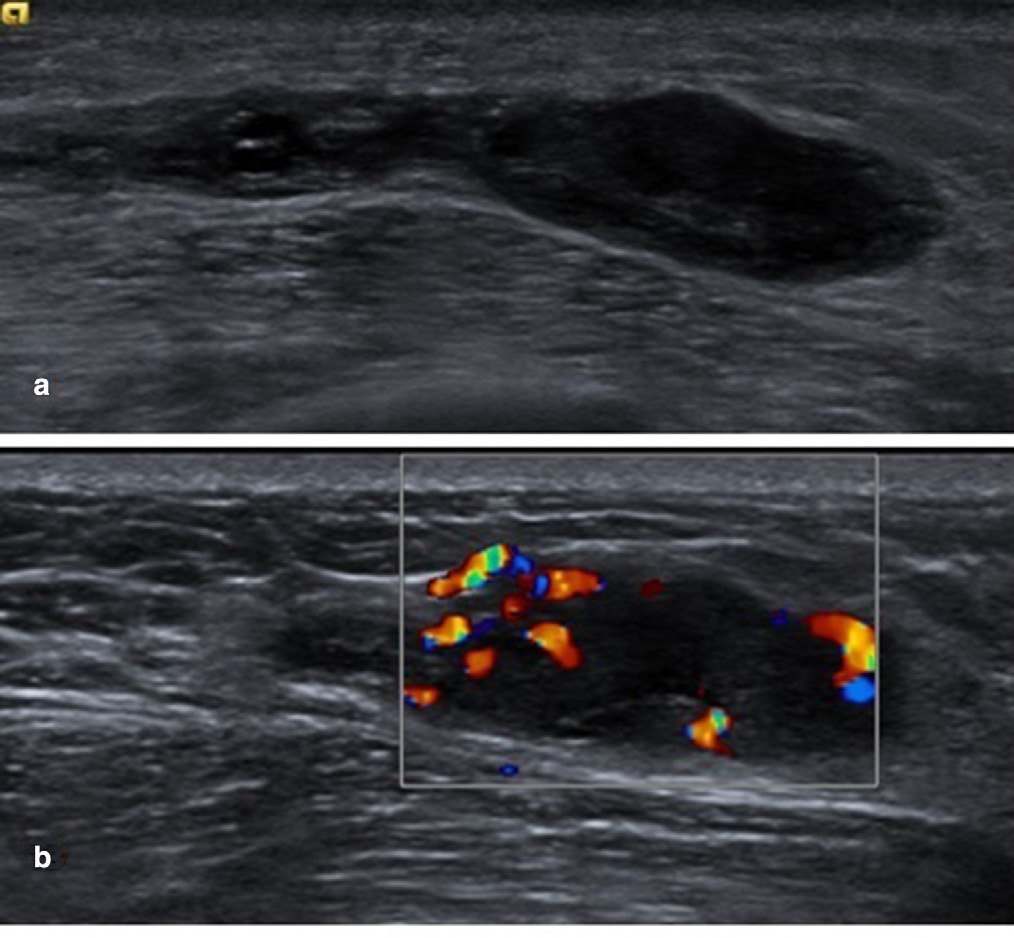
(A) Ultrasound images of the residual solid component following aspiration. (B) The presence of vascularity in the residual solid component is a worrying feature.

At her pre-surgical appointment, 6 weeks later, the mass had again increased in size and was uncomfortable. Ultrasound again identified a complex cystic lesion (U4) ; 40 ml of blood-stained fluid was aspirated and cytology results revealed high-grade atypical cells. This immediately prompted a further visit with re-aspiration of residual fluid, a further six biopsies (6 × 14G) sampling through the collapsed cyst bed and a marker clip inserted (Figure 3). The histopathology revealed invasive carcinoma, no special type (NST) , Grade 3 (B5b), oestrogen receptor (ER) positive (6/8), progesterone receptor (PR) weakly positive (5/8), Herceptin Receptor 2 (HER-2) negative. There were no abnormal lymph nodes in the right axilla.

**Figure 4.**
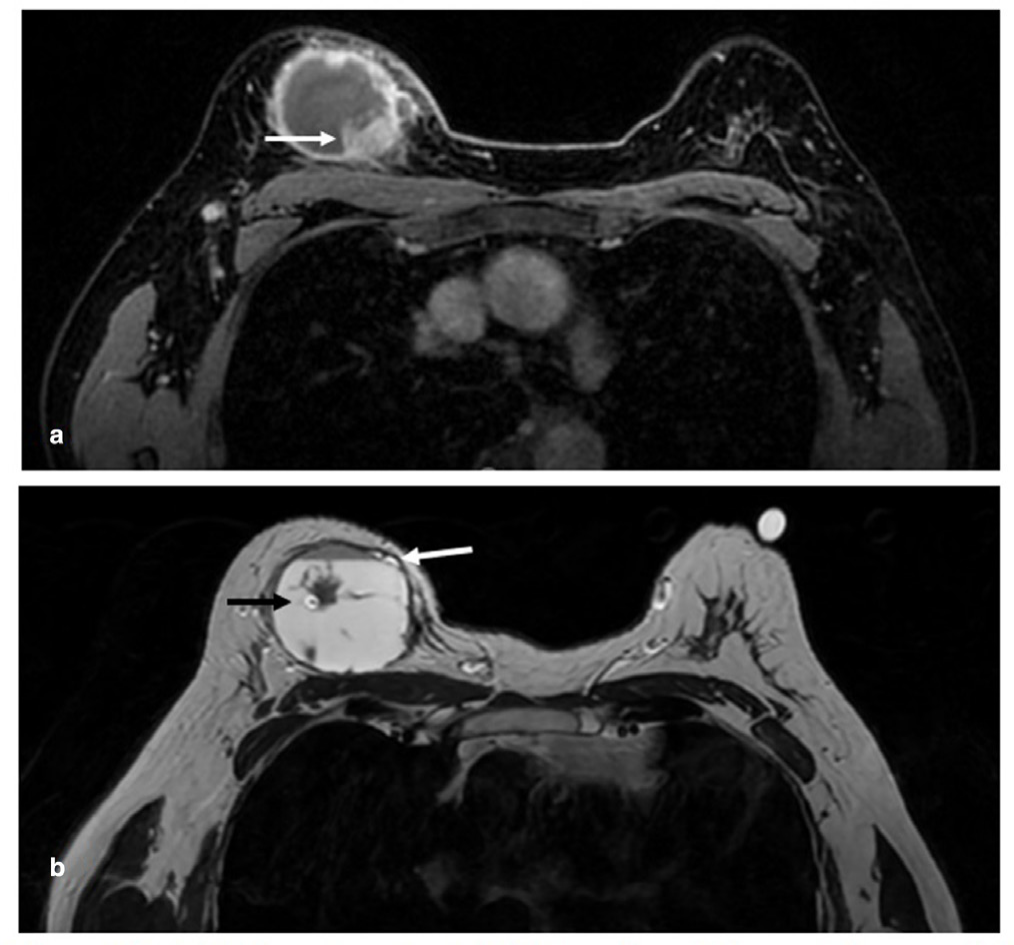
(A) Axial T1 post-contrast MRI showing a 50 mm cystic mass with a solid intracystic nodule in the right superior breast (white arrow) corresponding to the biopsy-proven malignancy. (B) *T*_2_ weighted image demonstrates high signal fluid with a fluid–fluid level (white arrow) of intermediate signal indicating blood products. The patient is imaged prone, hence the blood product signal is anterior. The marker clip is seen centrally (black arrow).

An MRI was performed for local staging which demonstrated a 50 mm cystic mass with an irregular internal thickened wall, fluid signal centrally but a solid intracystic nodule in the right superior breast with Type 2 enhancement.(Figure 4).^3^ Contiguous non-mass enhancement was seen inferior to the mass with benign Type 1 kinetics thought to be background enhancement.

Subsequent ultrasound fan biopsy through the non-mass enhancement confirmed benign changes.

CT staging of the chest, abdomen and pelvis did not identify metastatic disease.

## Treatment

The patient underwent a wide local excision and sentinel lymph node biopsy. Final histology confirmed a T3, N0^4^ Grade 3 NST with focal squamous differentiation. She underwent adjuvant chemotherapy, radiotherapy and extended endocrine treatment.

## Discussion

Complex breast cysts are defined as cysts with thick walls, thick septae, intracystic masses or other discrete solid components.^3^

Doshi et al^5^ adapting criteria previously described by Berg et al^6^ classified these into four types based on their sonographic features. Type 1 lesions show a thick outer wall, internal septa, or both. Type 2 masses contain one or more intracystic masses. Type 3 masses have mixed cystic and solid components, and are at least 50% cystic. Type 4 masses are at least 50% solid. Complex cystic breast masses have a substantial chance of being malignant with malignancy reported in 23–31% cases.^5,6^

The benign causes of complex cystic lesions include abscesses, apocrine metaplasia, inflamed or ruptured cysts, fat necrosis and haematoma. Short follow-up (2–3 months until resolution) of a thick-walled cystic lesion may be appropriate in the clinical setting of trauma or signs of infection.

The most likely malignant tumours to present as complex cystic soild masses on ultrasound are invasive ductal carcinoma (IDC) and ductal carcinoma *in situ*.^5^ If IDC presents as a cystic mass or a mixed solid cystic mass this confers a higher malignancy grade on histology.^7,8^

Berg et al reported that 18 of 79 complex cystic masses proved to be malignant. Of the 23 cystic lesions with thick walls and thick septations, 7 were malignant of which 6 (86%) were Grade 3 IDC.^6^

As opposed to complex cysts, complicated cysts are very common sonographic findings and the majority are benign (<2% chance of malignancy).^5^ Complicated cysts should never demonstrate internal vascularity at Colour Doppler interrogation. Since ultrasound is operator-dependent, optimising the image is paramount and together with the application of compound imaging and harmonics, Colour Doppler may help differentiate benign complicated cysts from malignant cystic-appearing masses.^9^

In this case, the initial history of trauma was misleading although the imaging was concordant with appearances of a haematoma on ultrasound. Colour Doppler was not used at this initial visit which may have helped identify suspicious vascularity. The patient re-presented to the breast unit due to refilling of the cystic mass. Any enlargement of such a lesion is concerning for an underlying malignancy and should prompt biopsy.^10^

The diagnosis was delayed as two initial core biopsies returned benign results. Reviewing the images, the needle is seen to traverse through the solid part of the lesion in both instances which highlights the inherent challenge in representative sampling. Complex cystic masses are challenging to biopsy because the technical difficulty is directly related to the presence of a fluid component. Aspiration of the cystic material and its collapse during biopsies can make the associated solid component imperceptible or hardly perceptible, and therefore more difficult to sample with certainty.^9^ It is paramount that if there is partial or complete collapse of the lesion, a post-biopsy marker should be left at the site of the biopsy.^9–12^

Image-guided core needle biopsy is preferable to fine-needle aspiration cytology of breast masses because of superior sensitivity, specificity, and diagnostic accuracy,^9–12^ although Doshi et al report that FNA may be performed in complex cystic masses particularly if the presence of a true solid component is in question on the basis of ultrasound findings.^4^

The MRI performed for local staging identified a malignant complex mass with irregular enhancement of the mass and the intracystic nodule. In retrospect, if MRI had been performed earlier as a problem solving modality, we would have come to the diagnosis sooner. Other authors have also suggested MRI as a sensitive problem solving modality in indeterminate breast lesions.^13^

High-grade cancers, papillary and mucinous breast primaries can all have cystic components.^5–7^ Metastases from other tumours to the breast can be well-defined, rounded, hypoechoic, may have posterior acoustic enhancement and therefore be misleading for a cystic lesion. Colour Doppler is invaluable to identify flow within these lesions.^5–9^ The finial pathology in this case had squamous degeneration which may have been a factor in the lesion’s unusual radiological appearance as squamous malignancies can have cystic components.^14,15^

Lastly, the importance of radiological–pathological concordance cannot be overemphasized. In this case, the diagnosis was elusive on two initial core biopsies, however as the lesion reaccumulated with fluid with no clinical reason, the level of suspicion for malignancy was high and therefore further biopsies and cytology was performed.

## Conclusion

This complex case identifies the pitfalls of diagnosing complex cystic lesions and demonstrates that if there is a high suspicion of malignancy then repeated biopsy and further diagnostic imaging is needed.

### Learning points

- A history of trauma may be misleading especially if the sonographic features point to a more sinister diagnosis: thickened wall (>0.5 mm), thick septae (>0.5 mm), presence of a solid intracystic nodule at second presentation and vascularity within the septae. It is important to be aware of these features that identify a complex cystic mass as the incidence of malignancy in these lesions is substantial (23–31%).
- The importance of clinicoradiological–pathological concordance cannot be overemphasized. There is a differential for complex cystic masses which includes infection, haematoma, fat necrosis, primary and secondary malignancies, hence clinical concordance is needed with further diagnostic work-up.
